# Perspectives of Vietnamese, Sudanese and South Sudanese immigrants on targeting migrant communities for latent tuberculosis screening and treatment in low‐incidence settings: A report on two Victorian community panels

**DOI:** 10.1111/hex.13121

**Published:** 2020-09-12

**Authors:** Chris Degeling, Stacy M. Carter, Katie Dale, Kasha Singh, Krista Watts, Julie Hall, Justin Denholm

**Affiliations:** ^1^ Australian Centre for Health Engagement Evidence and Values School of Health & Society University of Wollongong Wollongong NSW Australia; ^2^ Victorian Tuberculosis Program Melbourne Health at The Doherty Institute for Infection & Immunity Melbourne VIC Australia; ^3^ Department of Microbiology and Immunology University of Melbourne Melbourne VIC Australia; ^4^ Victorian Infectious Diseases Service Melbourne Health at The Doherty Institute for Infection & Immunity Melbourne VIC Australia

**Keywords:** Australia, deliberative methodologies, latent tuberculosis, migrant health, population screening

## Abstract

**Background:**

Tuberculosis (TB) elimination strategies in Australia require a focus on groups who are at highest risk of TB infection, such as immigrants from high‐burden settings. Understanding attitudes to different strategies for latent TB infection (LTBI) screening and treatment is an important element of justifiable elimination strategies.

**Method:**

Two community panels were conducted in Melbourne with members of the Vietnamese (n = 11), Sudanese and South Sudanese communities (n = 9). Panellists were provided with expert information about LTBI and different screening and health communication strategies, then deliberated on how best to pursue TB elimination in Australia.

**Findings:**

Both panels unanimously preferred LTBI screening to occur pre‐migration rather than in Australia. Participants were concerned that post‐migration screening would reach fewer migrants, noted that conducting LTBI screening in Australia could stigmatize participants and that poor awareness of LTBI would hamper participation. If targeted screening was to occur in Australia, the Vietnamese panel preferred ‘place‐based’ communication strategies, whereas the Sudanese and South Sudanese panel emphasized that community leaders should lead communication strategies to minimize stigma. Both groups emphasized the importance of maintaining community trust in Australian health service providers, and the need to ensure targeting did not undermine this trust.

**Conclusion:**

Pre‐migration screening was preferred. If post‐migration screening is necessary, the potential for stigma should be reduced, benefit and risk profile clearly explained and culturally appropriate communication strategies employed. Cultural attitudes to health providers, personal health management and broader social vulnerabilities of targeted groups need to be considered in the design of screening programs.

## 1 INTRODUCTION

The World Health Organization's (WHO) *End TB Strategy* aims to radically reduce the global incidence of tuberculosis (TB) by 2035 as a precursor to elimination.^1^ An adaptation of the End TB Strategy for low‐incidence settings provides an action framework for accelerating efforts towards TB elimination in these settings.^2^ Most cases of active TB disease in low burden countries such as Australia are caused by the reactivation of previously latent TB infection (LTBI).^3^ People with LTBI does not have symptoms, cannot transmit the infection, and, thereby, pose no immediate risk to others.^4^ Rather than being a stable state, LTBI is a spectrum from viable organisms actively replicating in a host to a status where the infection has been cleared or rendered ‘quiescent’.^5^ Therefore, the defining feature of LTBI is that it is not an active disease, but is a state of risk for developing TB disease in the future. This distinction has important epidemiological, socio‐cultural and ethical dimensions.^6^, ^7^ For most people with LTBI, the risk of developing active disease over their lifetime is low, with the risk of reactivation being dependent on their age and the time since infection.^8^ Consequently, LTBI is both a potential disease and an inconsequential infection in the vast majority of people who carry the mycobacteria.

Australia has agreed to establish and work towards a set of predefined targets, as recommended by the WHO’s *Framework towards tuberculosis elimination in low‐incidence countries*.^9^ In response, the Australian National Tuberculosis Advisory Committee (NTAC) has formulated a new *Strategic Plan for TB Control* that positions diagnosis and treatment of LTBI as a pathway to TB elimination in Australia.^10^ Recent migrants (<2 years) from low‐ and middle‐income countries are at substantially higher risk of active TB than non‐migrants.^11^ The epidemiological evidence indicates LTBI screening should target groups who are at highest risk of TB infection, such as immigrants from high‐burden settings.^12^ The cooperation of these affected communities is essential if LTBI screening and future TB elimination are to occur. In a qualitative study, Australian providers reported that migrant groups have difficulty understanding LTBI and can perceive LTBI screening as discriminatory.^13^ Australian TB programs are beginning to consider the implications of the elimination agenda for how TB services are provided in their respective jurisdictions.^14^ Yet the socio‐cultural dimensions of targeted LTBI screening have not been comprehensively assessed in Australia.^15^, ^16^

In this paper, we report on two community panels, formed of members of the Vietnamese and Sudanese and South Sudanese communities who live in Melbourne, Australia. Panel members were asked to consider and provide recommendations on what policy options for targeted LTBI case‐finding and treatment were seen as feasible and accepted as legitimate and fair. TB is a disease commonly associated with high levels of misunderstanding and social stigma in Vietnam^17^, ^18^ and Sudan.^19^, ^20^ Evidence also suggests that experiences of health care in an individual's country of origin influences patterns of post‐migration health service utilization.^21^ In Vietnam, for example, a complex set of beliefs and attitudes to TB treatment service providers can undermine treatment adherence and effectiveness. State‐provided TB health services in Vietnam are perceived by Vietnamese citizens as being too rigid, authoritarian and unable to respond to the needs of individuals; especially a preference for treatment flexibility and privacy.^18^ In Sudan, the prolonged period of ongoing civil conflict and political instability has had significant implications for the TB burden, and for TB control strategies, with large numbers of displaced, marginalized populations relying on weakened health infrastructure and an insufficient volume of health personnel.^22^ Reports suggest that many refugees and migrants from Africa do not prioritize engaging with healthcare providers in Australia,^23^ or other comparable high‐income countries.^24^

The intersection of migration and TB service provision has been identified as a determinant of the success or otherwise of the *End TB Strategy*.^25^ Current Australian policy is to pre‐screen migrants for active TB prior to obtaining travel approval.^26^ Because of their elevated risk of disease activation, children under 11 are tested for LTBI during this process, but LTBI testing is not included in other standard immigration pathways. Refugees arriving in Australia have alternative pathways not involving immigration medical examination and have existing recommendations for post‐arrival screening that includes LTBI. As TB programs begin to pursue elimination, key decisions need to be made as to the most appropriate setting for the LTBI testing of migrants to take place, and how best to communicate with potential participants to inform them about the potential benefits and risks of LTBI screening. Whether LTBI screening was conducted pre‐migration as a mandatory part of standard immigration processes or provided as a non‐mandated service after arrival in the new country would distribute the burdens of testing differently. Similarly, different communication strategies aimed at raising awareness about the elevated incidence of LTBI among migrants have different social risks and levels of effectiveness. For example, community‐specific campaigns (in non‐English language media), and English language posters and leaflets targeted to geographic areas associated with specific migrant communities may not penetrate to reach everyone who might benefit from participation. In contrast, a broad advertising campaign to improve LTBI testing uptake could reach migrants who are no longer closely connected with their cultural community, but increase the risk of racial vilification and public stigma because of reach to non‐target audiences. More established members of migrant communities in new host countries are key stakeholders to recent immigrants and can influence their knowledge, attitudes, perceptions and behaviours.^27^ Against this background, experience shows that effective targeted population screening depends on the alignment of the program with stakeholder values,^26^ and perceptions of the benefits and harms of participation. Involving members of a targeted community in a high‐quality dialogue about key issues such as these can guide program design, leading to increased support for the resulting policy and greater service or program utilization. This project is part of a larger implementation study conducted and funded by the Victorian Department of Health and Human Services to assess the feasibility and impact of shifting to a policy of TB elimination in this Australian State.^12^, ^15^

## 2 METHODS

### 2.1 Design

In a community panel project, a group of community representatives meets for 2‐5 days to carefully examine an issue of public significance.^28^ We convened 2 community panels, each lasting 2 days. The panel, usually consisting of 10‐14 individuals, serves as a microcosm of their broader community.^29^ Drawing on deliberative methodologies such as citizens’ juries, community panels are a useful tool for educating and engaging key populations in health policy decision‐making. In this instance, gathering views on potential advantages and problems in delivering a TB elimination plan, discussing and debating different possible weighting of community values. Similar methods have been used in Australia and elsewhere to consider issues surrounding infectious disease control and prevention.^30^ To be considered robust and reliable, deliberative processes must (at a minimum):

1. Provide participants with balanced factual information;
2. Ensure that a sufficiently diverse range of potentially conflicting, minority and marginal perspectives are considered; and
3. Create opportunities for free and open discussion and debate within and between community members and researchers or policy actors, or both, to challenge and test competing claims.^31^

The method assumes that people can think rationally and change their views should the evidence warrant it. Community panels are usually directed to consider a specific issue—typically formulated as a set of questions.^32^ They hear from a variety of expert witnesses and are invited to deliberate together on the issue. Panels provide evidence of public values and the likely acceptability, and perceived legitimacy, of different policy alternatives to assist policymaking.^33^

A panel of 10‐14 people cannot statistically represent their entire community. But it is possible to derive a sense of what an informed community would advise from a smaller group who are given factual information and time to deliberate.^34^ Community panel participants are recruited to capture the diversity of experiences and backgrounds in a community, and the deliberation processes organized to redress power imbalances as much as is feasible.^35^ When conducted in this way, community panels can capture and reflect key community concerns and arguments about current and proposed policy directions—that is, what should be done to address a specific issue.^28^

#### 2.1.1 Recruitment and selection

We contracted an independent professional research service to recruit panel participants. The recruitment company contacted potential participants using randomly generated list‐based samples of mobile and fixed‐line telephone numbers located in specific geographic areas, a targeted social media advertising strategy, and passive snowball sampling through community networks. This initial recruitment and screening produced a pool of potential panellists, with demographic and other information. The panellists were then selected purposively from the pool, with the final composition of each panel determined by individual availability and eligibility. Each panel was selected to promote an approximate 50:50 gender split and ensure a range of ages and socio‐economic diversity within each panel. Panellists were remunerated for their time including covering travel expenses if needed.

#### 2.1.2 Participant characteristics

Both panels were comprised of participants of mixed genders and ages (Table 1). The Vietnamese panel was skewed towards younger, male participants living in postcodes with higher levels of social and economic advantage according to the Socio‐Economic Indexes for Areas (SEIFA). Vietnamese migrants are a more established population group in Australia, with well‐established social networks, community organizations and language resources (eg Vietnamese radio and newspapers).^15^ Vietnamese panel participants were predominantly second‐generation migrants (except for 2 recent migrants). The Sudanese and South Sudanese community panel was skewed to younger participants with lower levels of educational attainment than the national average living in postcodes with lower levels of social and economic advantage according to the SEIFA index. The Sudanese and South Sudanese panel was entirely comprised of recent migrants—many of whom had come to Australia as refugees in recent years. Both panel events were held at a commercial conference venue in central Melbourne to make it easier for panellists from different localities to attend. The over‐representation of younger members of both community groups on the panels may be an artefact of the unwillingness of older members of the Vietnamese, Sudanese and South Sudanese communities to travel out of their local areas.

**Table 1:** Characteristics of panel participants

	Panel 1 Vietnamese (n = 11)	Panel 2 (South) Sudanese (n = 9^b^)
Age (y)		
18‐34	7	6
35‐54	4	2
>55	0	1
Gender		
Male	7	4
Female	4	5
Highest educational attainment		
High school	3	5
Trade/diploma	3	1
Bachelor degree	3	3
Postgraduate degree	2	0
Socio‐economic status of suburb^a^		
Low	2	5
Middle	3	2
High	6	2

^a^ Based on Socio‐economic Index for Area (SEIFA).

^b^ 1 participant unable to attend on Day 2 because of a family emergency.

#### 2.1.3 Procedures

**Figure 1:**
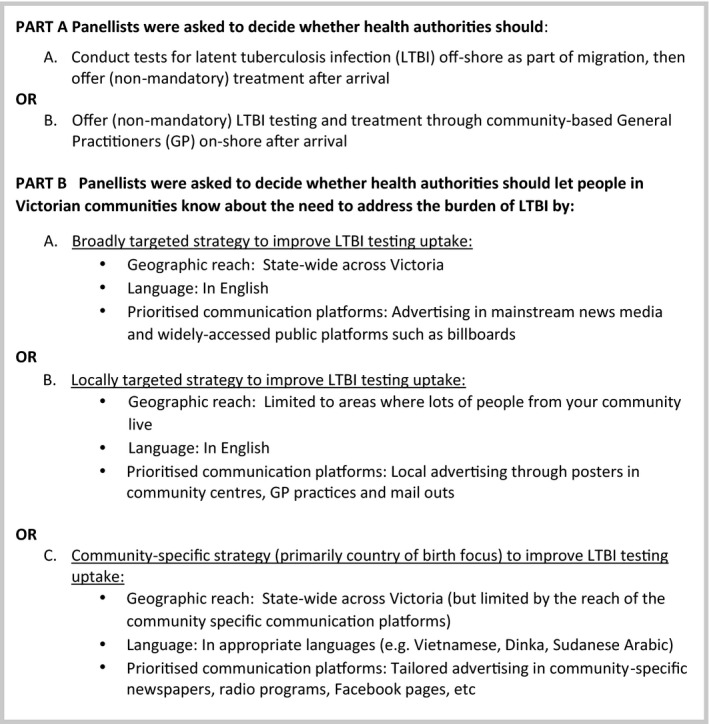
The questions posed to panels

Community panel participants were asked to consider and respond to the questions in Figure 1. Each panel commenced with an orientation session to introduce the process, the questions for consideration and to obtain consent. Panel Day 1 focused on understanding: basic tuberculosis biology (active TB and latent TB infections); the individual and population impacts of tuberculosis infection in Victoria for Vietnamese or Sudanese and South Sudanese communities, respectively; different interventions to manage tuberculosis disease and latent tuberculosis infection risks; and, different community communication and education strategies.

Four experts were selected based on their institutional roles, experience and expertise. They provided the panels with balanced and factual information supporting different perspectives on the potential impacts, benefits and costs of different case finding and communication strategies (Table S1). Each appeared in person, and each presentation ran for approximately 25‐30 minutes, including a question and answer session. After each presentation, panellists took part in structured stimulus and engagement activities to enhance their opportunities to make sense of and contextualize the information provided to them. These stimulus and question and answer sessions allowed panellists to cross‐examine the evidence and opinions presented. Because of their contribution to the design and conduct of this study, all four of the experts are authors on this paper.

For the first hour of Panel Day 2, panellists participated in a researcher‐facilitated discussion to reflect on and debate the evidence presented and their views on LTBI screening options. Panellists then deliberated for an hour, without researchers being present, to reach a verdict on the questions posed. The verdicts, underpinning reasoning and dissenting views, were then reported to the research team in a final facilitated feedback session.

### 2.2 Data collection and analysis

The two deliberative panels are the units of analysis in this study. All panel deliberations (facilitated and un‐facilitated) and expert question and answer sessions were audio‐recorded and transcribed. To track changes in the positions held by individual panellists, participants completed an anonymous ballot at 3 time‐points during panel proceedings: (a) after they had had time to consider the experts’ evidence (late Day 1); (b) after reflecting on it at home overnight (early Day 2), and (c) after the deliberation and delivery of the verdict at the end of Day 2. During the final session, a research facilitator recorded the verdict and reasons on a flipchart. Each point was reviewed by the panel to ensure accuracy. In what follows, we provide a summary of panellists’ descriptions of the rationale and reasoning that underpinned their responses to the questions asked of them.

Ethics approval for this study was received from University of Wollongong Medical Human Research Ethics Committee (approval number 2019/299).

## 3 RESULTS

### 3.1 PART A—pre‐migration or post‐migration LTBI testing

The Vietnamese and Sudanese/South Sudanese Community panels both voted in support of introducing mandatory LTBI testing to pre‐migration processes by consensus verdicts. Table 2 shows that support among the Vietnamese panel for the pre‐migration testing strategy was consistent across the weekend. For the panel comprised of members of the Sudanese and South Sudanese communities, the balance of the vote changed throughout, with support swinging definitively behind the pre‐migration testing strategy after panellists participated in deliberation. Table S2 contains illustrative quotes from the panels’ discussions.

**Table 2:** Part A deliberations

	Panel #1 Vietnamese (n = 11)		Panel #2 (South) Sudanese (n = 9^a^)	
	Pre‐migration strategy	Post‐migration strategy	Pre‐migration strategy	Post‐migration strategy
#1 Saturday pm After evidence delivered	11	0	6	3^a^
#2 Sunday am After reflection overnight	11	0	6	2
#3 Sunday pm After deliberation	11	0	8	0

^a^ 1 participant unable to attend on Day 2 because of a family emergency.

#### 3.1.1 Pre‐migration LTBI testing strategy

The key reasons both groups gave for supporting the pre‐migration strategy was that LTBI testing of people coming to live in Australia would be mandatory if incorporated into standard immigration processes. Both groups were concerned about the voluntary nature of post‐migration testing, reasoning that some people would not be tested and cases of otherwise preventable disease would be missed. Both groups held the view that new migrants would be understandably distracted with a range of other priorities as they adjusted to life in a new country. The Vietnamese panel told us that making the test mandatory was also a way of making it convenient and ordinary which would help to reduce the stigma of a LTBI diagnosis in their community. The saving for Australian taxpayers in making people wanting to come to Australia take (and pay for) the LTBI test as part of migration processes was seen as being more cost‐efficient. In contrast, many of the participants in the Sudanese and South Sudanese panel came to Australia through the refugee migration pathway, so convenience was not so important. For them, the extra cost to the migrant of the pre‐migration testing strategy was of some concern, but the group still wanted testing to be mandatory. They held it was better to protect their communities from imported disease as part of the process of people coming to live in Australia.

#### 3.1.2 Post‐migration LTBI testing strategy

Both groups saw some value in the GP‐based post‐migration LTBI testing strategy. Participants were of the view that involving local community‐based health services in LTBI case‐finding and treatment would help to raise awareness of the risks and burdens of LTBI among effected groups living in Australia. However, they foresaw that a lack of urgency and language and access barriers could limit testing uptake. Members of the South Sudanese and Sudanese panel also raised concerns about new migrants from their countries of origin, having lived through significant conflict, were not used to having routine health check‐ups. Both panels also expressed concerns that widely accessible communication about a post‐migration testing strategy might potentially increase the risk that migrant groups would experience further stigmatization in Australia. The public may come to associate migrant groups with specific infectious risks, and this knowledge may fuel outrage about the cost of the LTBI testing and treatment programs.

#### 3.1.3 Conditions on support for the verdict

Both groups placed conditions on their support for LTBI testing in a migrant's country of origin. To minimize the potential for alarm, clear information must be provided at the point of testing about what LTBI is and why the test is necessary—emphasizing positive aspects of your new country looking after your health and that of your family. For reasons of trust, it was also important to the panels that the treatment of any people identified as having LTBI must be undertaken in Australia. LTBI status must not in any way become an impediment to migration. Notably, the need for trust extended in both directions. Both groups lacked confidence in medical services in their countries of origin—both in terms of capacity and, for the Vietnamese group in particular, the potential for corruption among healthcare providers. The Sudanese and South Sudanese panel told us that LTBI treatment for those found to be positive should be compulsory. Crowding and poorer housing, health status and stress on arrival in Australia meant that new migrants were at higher risk of developing active TB disease and spreading infection. Groups felt that people who tested positive for LTBI in their country of origin should be bound by a health undertaking to complete preventive treatment on arrival in Australia to protect the local migrant community, who share these risks, from imported disease. Because all TB treatment in Australia is free for the patient (independent of whether they are citizens, residents, migrants or refugees), concerns about direct treatment costs were not part of panellists’ discussions and deliberations.

### 3.2 PART B—preferred communication strategies

The panels were also asked to consider how Victorian health services should seek to engage with migrant communities on the need to address the burden of LTBI. Acknowledging the strong preference of both groups for testing in the country of origin, we asked the panels to provide recommendations on appropriate strategies to inform their community in Melbourne about LTBI and the opportunities for testing and treatment in Australia. In coming to a final verdict, each panel member was allowed two votes in support of their favoured approaches to communication so that we could assess the acceptability, or otherwise, of different combinations of strategies. This scoring system means that the highest score a specific strategy could receive was 11 for the Vietnamese panel and 8 for the Sudanese and South Sudanese panel. Table 3 indicates that there was strong support in both groups for a community‐specific communication strategy including tailored messages in appropriate languages and on community‐specific radio and social media platforms about LTBI risks, testing and treatment. The key reasons were because this strategy would be more likely to reach people who were not proficient in English, while also working to address intra‐community stigmatization by providing a platform for education about LTBI and TB. However, the panels reached different conclusions as to the acceptability and effectiveness of combining this strategy with broad and/or locally targeted campaigns to raise awareness in their communities in Victoria.

**Table 3:** Part B deliberations

	Panel #1 Vietnamese (n = 11)			Panel #2 (South) Sudanese (n = 9^a^)		
	Broad strategy	Geographic strategy	Community strategy	Broad strategy	Geographic strategy	Community strategy
Final vote Sunday pm After deliberation	3	10	9	5	3	8

^a^ 1 participant unable to attend on Day 2 because of a family emergency.

#### 3.2.1 The Vietnamese panel

As well as a non‐English language community‐specific campaign, the Vietnamese group strongly favoured the use of locally targeted awareness programs in areas where lots of people from their community lived. The outcome of combining these strategies would be a place‐based approach to communication. On this, it is worth noting that participants in the Vietnamese panel were almost entirely second‐generation (except two recent migrants) which modified their views to the extent that many of them identified themselves as being Australian. The group saw the value of a broad English language and mainstream media awareness‐raising campaign because it would act as a reinforcement, reminder or both of more targeted messages. But concerns about vilification of migrant groups with high LTBI burdens led them to recommend that it should not be implemented unless it could be done in a way which did not identify any specific country of origin. This concern about the risks of racial stereotyping extended to how community‐specific messages were implemented. The group took the position that any non‐English language awareness‐raising campaign tailored to Vietnamese and other migrant communities should all be rolled out simultaneously so that no group feels they are being singled out and unfairly targeted.

#### 3.2.2 The Sudanese and South Sudanese panel

In contrast, the Sudanese and South Sudanese panel were much more divided with most participants preferring to enhance community‐focused awareness‐raising with a broad English language non‐targeted public health messaging campaign. The Sudanese and South Sudanese panel was entirely comprised of people who were born overseas, many of whom had come to Australia as refugees in recent decades. They told us that this lived‐experience of migration made them acutely aware of the potential for harmful discourses and vilification. The reasons given for supporting or rejecting non‐English language community‐specific communication strategies were almost identical to those given by the Vietnamese panel. Ultimately, there was unanimous support for the community‐specific strategy because it was important to engage with leaders: the structure of the community is hierarchical with members placing most trust in community leaders to provide information and advice on what member should do. They reported that Sudanese and South Sudanese people mistrust external organizations because of past negative experiences of apparently well‐intentioned service providers. To minimize the potential for social harms and in order to be effective, communications need to come from trusted sources. Respected leaders are the trusted gateway to reaching out across all the different parts of these communities.

Finally, both panels were informed about the role of BCG, common treatment regimens for LTBI, and their potential benefits, harms and limitations, during the expert presentations. However, concerns about people being asked to take medication when feeling healthy to prevent the development of disease did not figure significantly in their subsequent discussions.

## 4 DISCUSSION

The Vietnamese and Sudanese and South Sudanese panels involved in this study were highly supportive of testing migrant groups for LTBI, consistent with previous research indicating that new migrants accept most forms of infectious disease screening.^36^, ^37^ The key concern shared by the panels was the possibility that LTBI screening could lead to social harms such as stigma, both within their own communities, and against their communities from other Australians. The strong preference for testing to occur pre‐migration was not simply about imposing the burden and costs of testing onto others, but was seen as a way of making sure that all new migrants were tested for LTBI, and that these activities were performed in a setting removed from the view of the broader Australian public. Despite differences between the groups in perceptions of the degree to which they identified themselves as being part of Australian society, both panels also expressed uneasiness about the potential for any communication strategy to identify and single out specific cultural groups—increasing the risk of negative public discourses, racial vilification and social stigma. Pre‐migration testing was thereby seen as a means to mitigate many, if not all, of the social risks of targeted LTBI screening. Failing that, and acknowledging that members of migrant communities who had been residents for some time could also benefit from LTBI testing, the recommendation was that any broad and widely accessible communication strategy about the need to enhance LTBI case‐finding efforts in Australia should be generic and not explicitly connect the condition to any particular migrant community.

These concerns seem justified as several studies suggest that TB control measures and representations of migrants in media reporting of TB are implicated in the stigmatization of migrant groups.^38^, ^39^ It is likely that negative impacts of targeted LTBI case‐finding and treatment programs could be amplified in the Australian context as both historically and currently TB control has entwined immigration and public health policy while also serving as an arena for xenophobic political strategies.^40^ Public health discourses can also have negative effects causing people to become aware that they are members of a stigmatized group. Being labelled ‘at‐risk group’ for TB by the health service in their new country of residence can further magnify migrants sense of being ‘out of place’.^41^ To counter these impacts, and empower the local community to provide appropriate advice to recent migrants and their more established members, both panels emphasized the importance of involving community leaders in migrant health service planning and communication. Consistent with evaluations of migrant focused health programs in other settings, both panels highlighted that different cultural identities and migration histories meant that their communities functioned in ways that require nuanced and sometimes heterogeneous types of engagement between program managers, community leaders, and different socio‐demographic and ethnic groups within each community.^36^, ^42^

Negotiations and efforts to clarify who the LTBI screening service should be designed to benefit needs to be a central element of these discussions.^7^ Both groups who took part in the study expressed a strong preference for LTBI screening to occur pre‐migration because, from their perspective, off‐shore testing would maximize the benefits for incoming migrants while minimizing the impact on local migrant communities. Although it was not described in these terms, the groups wanted to balance effectiveness and risk of harm in communication by ensuring that the risk for LTBI and the benefits of testing and treatment were well understood by their community, but that this understanding was supported in a way that did not simultaneously promote stigmatization and discrimination.^43^ That post‐migration screening would reach fewer migrants was seen as a major limitation for this strategy, reflecting the importance the panels place on maximizing effectiveness but also to equity of access to health benefits. Previous qualitative studies in the UK suggest that the optimum approach in high‐migrant receiving countries is most likely to offer screening in a range of settings.^42^ While not the preferred strategy, opportunistic screening for TB and LTBI in primary care was acceptable to both panels involved in this study. Previous work in the UK suggests that testing for LTBI during GP consultations can be an effective non‐coercive strategy for increasing participation by high‐risk groups in post‐migration screening.^44^ Both groups noted that the provision of accessible and appropriate information to migrants was essential to testing acceptance (both pre‐ and post‐migration). In this context, the accessibility of the information is a function of its format and comprehensibility such that all users can access the content on an equal basis; and appropriateness means the information is correct and fits the goals of the communication. These recommendations are consistent with experiences of European TB programs which indicate coercion can be counter‐productive if it is accompanied by insufficient information and unable to provide valid arguments for why migrants should participate.^45^

Cognizant of the potential for pre‐migration screening to create new barriers for migrants, the groups also emphasized that their support for implementing off‐shore LTBI testing depended on the test not becoming an impediment for people wanting to come and live in Australia. Both of the panels sought to find a way to ensure that new migrants, migrant communities living in Australia, and the broader Australian public took on some burdens and received benefits from LTBI screening. On this, both panels explicitly emphasized that trust was an important requirement for appropriate service design and delivery for LTBI case‐finding and treatment. Medical services in Vietnam and Sudan and South Sudan were not seen as trustworthy, such that people found to have LTBI needed to be treated in Australia. Trust was also central to effective communication such that building on existing community relationships was also an important feature of the strategy for both groups. In theory, the principle of reciprocity can guide approaches that both promote the benefits of participating in LTBI screening and compensate participants adequately for the burdens of participation.^7^, ^46^ For the members of the migrant communities who took part in this study, offering free treatment from the Australian health system was seen as part of the reciprocal relationship between new migrants and their new country established by screening participation.

### 4.1 Data limitations

Members of the Vietnamese and Sudanese and South Sudanese communities living in Melbourne without a high level of English language fluency were effectively excluded from the study due to the use of English language in recruitment materials and during panel proceedings. For the Vietnamese group, in particular, older age groups were under‐represented such that participants spoke of and considered the needs of older members of their community, rather than these perspectives being represented by first‐hand accounts in discussions. A further limitation is that community panels are comprised of small groups of ‘engaged community members’ whose views may not represent the complete range of perspectives held within otherwise internally heterogeneous cultural groups. We did not systematically collect data about the amount of time each participant had lived in Australia. Rather individuals identified themselves during discussion as recent, first or second‐generation migrants, with many of the Vietnamese Australian participants having been born in Australia. However, because both panels were comprised of individuals with a range of ages, backgrounds and migration histories, and because both panels came to broad agreement on their preferred LTBI screening and health communication strategies, it seems likely that many of the issues and concerns raised by participants would be shared by members of the same cultural communities living elsewhere.

## 5 CONCLUSIONS

Migration is a varied process that has implications for both migrants and TB service providers.^25^ Policies on migration‐related TB screening vary considerably across low‐incidence settings indicating uncertainty concerning effective methods for migrant TB screening.^47^ Challenges faced by migrants such as communication problems, loss of social support, discrimination and acculturation can be aggravated by fear of a positive TB diagnosis.^48^, ^49^ There has been little prior research focused on the specific experience of new migrants and their views on ways forward.^36^ Our findings are not necessarily generalizable to other migrant groups or other national or health system settings. Nevertheless, as health authorities and TB programs in low‐incidence setting begin to plot a pathway to elimination, the current study highlights the critical importance and social value of incorporating a strong focus on community engagement and partnership with migrant organizations in both the design and implementation of acceptable and effective strategies for LTBI case‐finding and treatment in migrant communities.^50^

## CONFLICT OF INTEREST

Justin Denholm is the Director of the Victorian Tuberculosis Program. All other authors have no conflicts of interest to declare.

## AUTHOR CONTRIBUTIONS

CD designed the study, ran data collection and analysis processes, and led the drafting and revision of the manuscript. SMC contributed to study design, participated in data analyses and made significant contributions to the drafting and revision of the manuscript. KD, KS, and KW developed the evidence presented to panellists, participated in data collection and contributed to and revised the drafted manuscript. JH contributed to data collection and made significant contributions to data analysis and the drafting and revision of the manuscript. JD designed the study, participated in data collection, contributed to data analysis, made significant contributions to the drafting and revision of the manuscript.

## Supporting information

Table S1‐S2

## Data Availability

There are no data available for sharing because of conditions imposed by the Ethics approval under which this research was conducted.
