# Supplementary material for: Perspectives of Vietnamese, Sudanese and South Sudanese immigrants on targeting migrant communities for latent tuberculosis screening and treatment in low‐incidence settings: A report on two Victorian community panels
Source: Health Expect. 2020 Sep 12;23(6):1431–40. doi: 10.1111/hex.13121 (PMC7752196; doi:10.1111/hex.13121)
Supplement: Supplementary file 1 — Table S1‐S2 [file HEX-23-1431-s001.docx]

| **Supplementary Table 1:** Expert testimony provided to the Community panels | | | |
| --- | --- | --- | --- |
|  | **Expertise** | **Expert area** | **Data provided** |
| 1 | Tuberculosis, Infectious disease specialist | Clinician, tuberculosis disease control and prevention. | - the basic biology and impacts of tuberculosis infection (e.g. what it is, how the bacterium spreads, common symptoms, morbidity and mortality); - differences between active & latent tuberculosis infections (LTBI) - the threat tuberculosis poses to human health - public health surveillance systems - measures to prevent and manage tuberculosis disease incidence and spread |
| 2 | Epidemiology | Public health – Tuberculosis epidemiology in Victoria | - how the tuberculosis epidemic has been managed in Australia - the impacts of migration in rates and risks of TB disease in Australia - evidence as to the burdens of latent tuberculosis infections in Greater Melbourne - evidence as to the burdens of LTBI among different migrant communities in Greater Melbourne |
| 3 | Tuberculosis, Public Health physician, Infectious disease specialist | Public Health, Tuberculosis disease control and prevention, Public policy | - the purpose and value of different strategies for LTBI case-finding and treatment in migrant communities in Victoria - the potential benefits, harms and costs of testing for LTBI in a country of origin during migration processes (pre-migration) - the potential benefits, harms and costs of testing for LTBI post-migration as a community-based intervention |
| 4 | Health social work, Community Engagement | Clinician, health science, community engagement | - the role of health communication in promoting good health outcomes - evidence and ethical perspectives on the potential benefits and harms of rolling out - a broad Victoria-wide but community nonspecific public communication campaign on LTBI - a geographically-specific public communication campaign on LTBI - a community-specific communication campaign on LTBI |

**Supplementary Table 2: Illustrative Quotes from Panel Discussions**

| **Theme/Topic** | **Panel** | **Illustrative Quotes** |
| --- | --- | --- |
| Perceptions of LTBI and TB | Vietnamese Panel | - Because I believe that everyone, mostly Vietnam is – they do know TB, what that does mean. Is that the young generation? It’s just like oh, is that what TB is? And then, some people, it’s is he going to die? They don’t know. But they know TB is quite serious and everyone is scared Pg 25 D2. - It is kind of scary because it’s latent TB and people mistake it for active TB. I feel like if we can somehow change the name of it to be more friendly as in sleeping or inactive TB or something, would distinguish the two things, so people wouldn’t get the perception that that’s actually active TB. D1-T4 pg 6 - I think that – I think we should change the name of latent to be – into something more friendly, as in like inactive or intransmittable or something like that. Because latent is not very in general knowledge and so D2 pg 130 - …., it would definitely depend on how active the person is regarding their health. Because some people might be like, if it’s latent. Other people might be more vigilant about it.DI-T3 Pg 17 |
|  | Sudanese & South Sudanese Panel | - They don’t know about TB. They don’t know TB but maybe some of them have got TB. As you say, the areas of concern are this and this but they don’t know. Even your brother maybe, you don’t know. T4 SMC Pg 7 - I know with me, like I’ve been careful for all my life, and I did not know TB. So if it was just me, I know there’s a thousand other people walking around that have not known what TB is. T4 CD Pg 13 - We only know one, even me I only know one TB, I didn’t even know about the sleeping TB. SD2 Pg 120 - We still – people feel fear that there’s a chance that I’m not going to get well with their treatment. But in Australia, they find it for me, I’m happy because I’ll be treated. T4 CD Pg 45 - It [LTBI screening] will bring access to the whole information T3 SWOT Pg15 - Everyone already through thought you are okay because you have been screened and everything is fine. T4 SMC Pg 15 - ... as soon as you arrive here, you will not stay by yourself. You will stay with family and young children that could be exposed or [00:52:06] take a time. Like, we don’t - if someone comes with TB, we wouldn’t know in the family, because you wouldn’t know, but they’re more exposed. So I think there’s more chance of it spreading T4 CD Pg 45 |
| Stigma - *how was stigma discussed and perceived. Drivers and harms* | Vietnamese Panel | - Mostly, I don’t know in Australia, but in my country, quite [00:45:53]. If they know you’re TB, they try to stay away. Because they know how it really infests. So they are – so to let them know how serious, because they already have some idea how serious, and have them to sort it out. Pg 25 D2 - And I will say this, with Vietnamese that can be quite judgemental. … they can be really scared and then, they can be judgemental and so, you have to equip them with the right information. D2 Pg 44 - But if they see you at actively going to a centre to get tested for TB, they will assume that oh, he might [00:20:26] or higher chance of exposure already. Yeah. D1-T3 Pg 21 - But if you have it onshore people might go, I don’t want other people to find out. I don’t want to. D1-T3 Pg 22 - I think their made feel like they’ve been demonised again for something they can’t control. DI-T4 Pg 9 |
|  | Sudanese & South Sudanese Panel | - but I guess with this one, that’s where like, the shame comes from, because if you go and be like, oh yeah, I have this sickness and we’ll all be like no, get away from me. So you’re like, I’m going to stay quiet and then, just by staying quiet and just taking time to even go to the hospital, you might affect someone along the way. T4 CD Pg 7 - Let’s just say a group of the white minority find out that the South Sudanese community is the number one people that have TB and then they found out. Like, we already experienced enough like,T4CD Pg5 - Yeah. But what (Anon panel member) is saying, for example…There’s always things happening already in the community, we’re all already isolated, so that – that’ll increase the issues that - - - T4CD Pg 5 - Like, even though it’s a disease that everybody could get, but then I guess, when like, some racist people, when they hear that about see, yeah, no, this is another reason why these people don’t belong here. They’ll just find any reason to [00:05:34] and blame us for it. Like, even if it’s not in our health [00:05:37]. T4 CD Pg 6 - oh, they’re animals and then they found out like, oh, you guys bring disease to our country. So I guess I’m just aware that fear comes from, because we’re already like – we didn’t do anything wrong, but then we get looked down upon like this is all your [00:04:56, ?fault?] everything you do is not right. So if we’re coming here with our sickness, people are going to be like see, this is exactly why I’m going to stay in my country. Like, no one should come to this country. T4 CD Pg 5-6 - … if it's – we’re using the Sudanese or Asians or Vietnamese then when people go in public places people see whoever, when they see Sudanese or Asians they say oh, tuberculosis, tuberculosis, like, you can see now what happened with the Sudanese youth or African gang issue, no matter when they see the person they said, okay, whenever something happens they say deport them, that’s the only word it comes, no matter what, deport, that’s the word they said SD2 Pg 103 |
| Perceptions of and attitudes to Health Service Provision  *Plus some relevant health behaviours findings* | Vietnamese Panel | - I think it’s good that you mentioned the chemist, ‘cause my parents have a great relationship with their pharmacist and their doctor and if [the pharmacist] says something that’s it; they’ll do it. Definitely. D1-T4 pg 5 - I’m curious, like, what’s the turn up of the participant rate of the people, like who were willing to go to the GP to be tested for that, because Vietnamese people are quite nonchalant regarding their health. D1-T4 pg 9 - They’ll take the risk. So, the statistics is one in four, so that’s about a 25% chance that you have it. I know people who would willingly not go to the GP, take that risk, oh it’s only 25%, you know. Even if I do catch it, I’ll know when I catch it. I can go to the GP, get medicated. I’ll be safe, there’s only a 5% chance that I die. They will take that risk, like 5%. D1-T4 pg 9 - Yeah, it’d be ideal to have a support service, so community health worker, bilingual, to divert the enquiries and not just have a kind of – at GP level, because often, that’s not accessible for some people. But just to – yeah, there’s a demand on the current bilingual GPs anyway, in our community. There’s only a handful of bilingual that are good ones. Pg 24 D2 - I think that was a trust thing. I don’t think the Vietnamese probably won’t trust the actual health system in Vietnam. I think they’ll trust a health system a lot more in Australia. Pg 101 D2 |
|  | Sudanese & South Sudanese Panel | - When I came to Australia what come in my mind is to go to school and health wise I wanted to be healthy, I want to go to doctors, I want to get treated so I can feel – I want to feel okay SD2 pg42 - …we have been burned – the whole community has been burned by the services, so we kind of don’t trust the services anymore. T4 CD Pg29 - I think where that fear comes from because there was a big floodgate of government funding where people were accessing to deliver services to the South Sudanese and a lot of people were just ticking off boxes, so they’re just grabbing people and just ticking off their boxes and they go and they’ll see an event, while they’ve – they’re forgetting that these people that are approaching them have genuine issue and they need a real support. So a lot of communities, so when we started a lot of people were saying, “Oh, are you one of that service that will come and just disappear the next day? You take my name and that will be the end of it.” So there's fear behind that - - - T4CD Pg 133 - ..what I found working with other services is that there's lack of cultural competency within, so I would – what I would think is to get training in regard to cultural competency because there are things that you would do and that would offend certain community but you’re not aware of it T4CD Pg129 - I wanted to bring this into your attention because we came from a war torn country, a lot of us don't do health, so health check-up, regular health check-up, especially in our community is one of the thing that is lacking. And this is one of – it's very important to bring this awareness and that the importance of having a health check-up because a lot of issues that are raising up, like cancer, it's actually a problem in our community because people don't do regular check‑up and then when they detect it it's too late, so it's not something we do as a community to go every year or every two years for a health check-up because we grew up in a – people came from a war torn country so health it was not a priority, where are you going to do it - - -T4CD Pg 135 - …we don’t have that orientation. When we came in, what we need to do, 12 months’ time, you’ve got to have like a 12-month check. Like, your personal kind of health check. So every 12 months you’ve got to have a health check to see what you have, so you can have an (? early prevention). We don’t have that information.T4CD Pg9 |
| Communications | Vietnamese Panel | - I’m second generation Vietnamese but if I watch a video, if they were Vietnamese, I would find more. I don’t know; it just tugs at me, I don’t know how or why, but it’s my mother language. I’m not completely fluent but I understand it and it has more of an emotional draw, when I hear things in language. D1-T4 pg 3 - It just depends on how you deliver the information. If you make people scared, they’re going to be scared. If you make people understand that there are risks but at this point you are not at a high risk, they’re not going to be as scared. It just depends on how the message is delivered. D1-T3 pg 21 - When you target a minority, I feel like you’re, targeting a minority is good and that, so some of it could be good, because you’re getting that community very aware of what they’re doing, you know, telling the Vietnamese people are most likely to get tuberculosis makes me feel like, oh I need to get checked out, before others who may feel like to them that, oh I’m getting singled out or I’m getting stigmatised that I maybe sick. D1-T4 Pg 3 - Yeah, to be fair, if you reach one of the generation, all these generations, they will pass down. D1-T4 Pg 5 - For certain people - if you communicate in the wrong way they would perceive it to be more contagious than it would be. DI-Final pg 4 - I was just thinking of my family, to begin with, and how comfortable they would feel receiving it; so their experience. As parents who can’t speak English. How reassured would they feel? And we talked about local doctors and chemists, they trust them. Trust is an important part of how they receive the information – they’ve got to trust the source DI-Final pg 5 - I think one of the important points that I hope that it comes across to people that latent TB would not be able to pass it on to other people. Because if you don’t consider that message as one of the key points then it would give the general population a false sense of perception that if they have latent they would be able to pass it on to others. D1-TFINALPg 9 - But if it’s raised like – if for breast cancer or something, and it’s like oh, give the ratio and how you could be affected by that, then I think it should be fine. But if it’s this state-wide, like generic message and then they highlight the community groups that are more likely to be affected by the disease, then it’s like the outer group and inner group, like tension between different communities. - - -pg 17 D2 - But I think it should be in areas where more partners or women, because when – you don’t want to hear a Vietnamese woman nagging, let’s put it that way. So if she comes home and she says, “You need to do this.” You’re going to do it, because the nagging itself alone would probably kill you, so you go okay, I’ll go do it. So your mum or your aunt or your grandmother says, “Have you done this? Have you done this? Have you done this?” And then, so you’re disrespecting your mum or you’re disrespecting, go and do it. Yeah, okay, I’ll go and do it. PG 45 D2 - And also, if you’re already at a hospital or a pharmacy or the doctors, you’re already there to be, you’ve acknowledged, so you’re already primed to go okay, I’m here because I’m sick. I’m here because I need help, and you’ve got something there that actually says here’s what could possibly help you. You’re more already primed to receive that information pg 49 D2 - You tell one rational Asian mum; the rest of the community will know. PG 51 D2 - So the chemist is very good starting point and university, so the kids come home and say, “Mum, Dad, have you seen this?”pg 4 D1 T4CD - My parents go to the market and find information about politics and news. D2 pg 132 - it’s also understanding, emotionally, how Vietnamese people take in information, and that’s the whole thing and how we communicate the things – it’s interesting, because - -I think it’s how we – how they actually inform the community, without scaring them, without bringing fear.D2 pg 65 - Like, it’s not just about your own health, but you can – it’s the safety of your family as well. Like, not passing it on to your relatives. D2 pg 120 - I think what about having people on the streets handing out pamphlets and actually talking to people to reduce their fear. So, health workers, social workers to hand out and say, here are the options that you do have. There’s no – this is a service, we’ll package it in a way where you – so, we’re providing a health service thing that you could benefit from and explain it that way to people and you can, once again, target events that they have around those. In Springvale or Footscray, you have all the Chinese New Year, you have the Moon Festival. You have these things where people are already there, and then, you can actually talk to them and hand out things and so, it’s just easier, rather – easier to just piggyback on an event that’s already done by the local community. And then, you’ve got the newspaper there. You had all the businesses there. You can pop posterise in their windows and things like that. - I really like this idea [locality-strategy], because essentially, increase the current credibility and the trustworthiness of the authority, because you set up a booth in an existing festival, then it’s really credible, people come to you and then ask for your advice D2 Pg 113-14 - Yeah, so you just incorporate it into – the thing about I think most patient’s families is like, we all eat together, we do certain activities together and if it’s discussed just amongst – in a very fluid way, I think you would be able to turn people around to it easier than if you came from an outside point of view, coming in, then it creates fear. Because of the – what I’ve learned is they’re quite superstitious, there’s this fear level, some people lack of education. So lack of education creates more fear in certain things. And a huge crossover between like, say my parents and I, communication issues, because I couldn’t speak Vietnamese very well. So if it’s done in an event where there was some – media in between, it’s talking to them both in Vietnamese and English, they could probably consume the information and take it in a lot easier. D2 pg115 - and somehow try to avoid to mention Vietnam is high-risk area and high-risk country like that, pg 116 D2 - I think it should – I mean, obviously, it must have different language, other than Vietnamese, because you don’t want to be targeted. So as long as you have many different languages, including English, people are not going to feel targeted. It’s just that the government and the healthcare system is trying to convey the information in a better way, for more people to read it. So yeah, it just to be, you know, like many languages and in English.pg 119 D2 - So if you get the right person to be with you and deliver that message, that the community – it’s about trust. So if you’ve got the right person to help – who’s supposed to deliver that message, it can be received. But if you can’t, it will just talk and that will be the end of it. Because [00:30:32] good and bad. We’re choosing. T4 CD Pg29 |
|  | Sudanese & South Sudanese Panel | - So you have to put in mind, it’s so hard to engage with our community. ..we’re a very close knit community and we’re very guarded so we don't – so it's to work on building trust and saying that I’m not doing this to just tick off my boxes and get my funding and disappear, I’m doing this because I’m genuinely concerned about the community and I want to bring this awareness and that message would be – it will take a minute but it will be received eventually T4DC Pg 131 - …don't just jump in, have knowledge around the community, who are the gatekeepers, because those are the people that would – will make your delivery of the message more effective instead of you doing it, you might not get anyone turning up T4DC Pg 131 - How this message can go out to the community, it is when the leaders of the community, because they are the ones who are calling the community, the men and women and also we have got youth leaders that can call the youth so that information can go right away into the community. So, through the ring leaders of the community. Not the [ordinary] members… T4 SMC Pg1 - Firstly, you contact the leaders, tell the programme and tell them there will be an advertisement. Don’t be surprised. It is concerning your community on TB…. Any person who is asking the leader, the leader says, I know. T4 SMC pg5 - The easiest way, the best way is through the communities and the community leader will know, the community leader should go for that. They are for youths, they are for women, they are for elders, this sort of group, they would know all these messages are linked to them, yes. Not through this radio and all that, not everyone is accessing all those things. Yeah. But the community is the way forward. T4SMC Pg9 - I feel like the elders are the ones that come talk to us, tell us what to do, so on and so on so, yeah, if anything it's the elders then you know [01:33:47], yeah - Here in Victoria, we have various languages and I think to me, the best way to tackle them even though is, it could be quite challenging is community based in terms of, because in our different communities we have got different leaders whom we choose to represent us in social and all those things. If there is anything that is coming up they call us and convey the message to all the people but not everybody will be there. Especially the young, they are not there all the time. That means it could go in a different way…. When it comes to social media, not everyone is accessing social media as well but what I think could be effective is group targeted using the leaders to convey messages and all that T4 SMC Pg 1 - …in the community, especially the young people, respect adults. Whatever the adults agree the majority of the youth or younger families would agree on because they’re, for example, Simon, if he says whatever, “This is what – this is good for us,” and he explains in the language people will understand it more. But if somebody came and just said, “Oh, this is what I think is better for you,” there will be some questions about what does that person know about us? So they, our community, listen to the message from withinT4CD Pg 128 - Anything that goes to for example to Melton, before it goes to shopping centres and all that, the leaders should convey that message before it goes there because you know, especially in Victoria there are these understanding because of South Sudanese or Sudanese crime and people and all that and if you just take those messages to those areas it would be perceived in a very different way. …….. Instead, you are taking the positive or it and they will just see the negative of that. That they are being targeted and it will just destroy the whole, if you go to the leaders first then they will give you their strategies to go about it and the message will go out very easily T4 SMC Pg5 - The community specific, I think the message will be received well and it will go to people that are targeted and I think there's also – because in regard to discrimination there's also that protection because it's not widely – not everybody knows, it's only that community does knows what’s going on and this is the action, so it could be the action would be taken in a little amount, in short amount, that’s what I felt, and it would be more powerful, it will be received well by the community, yeah. SD2 Silver Pg 15 - I think in my opinion it is better if you talk in general message because if you are say a certain group like South Sudanese, I think they are going to feel like you are targeting us. So, the general is better than, yeah. I think it is good to approach them. T4 SMC pg 6 - We say let it come as general then get the Sudanese or whoever migrant that are in the communities and pass your message. So, you need to generalise first, don’t use a certain group or Sudanese. We don’t want to be targeted. T4 SMC Pg 7 - So like that targeting you is also good, but then it’s not, because then everybody will look at you like you have this disease, all that.T4CD Pg33 - So I guess if you want that one on one targeted to Sudanese person, like most Sudanese communities, like African clinics where there’s an African doctor, where a lot of Sudanese people go there and then you can have a more targeted audience, like one in five Sudanese people – south Sudanese people have it. T4CD Pg31 - I guess you have to maybe think of approaching it in a different way. Like, in the community sense, but then more of in a private way, where you can go to a circle and learn about it and then, if you feel like you haven’t, there’s a place where you go where it’s private, you can talk about it and get what you need to know and then, you know? T4CD Pg 7-8 - Because obviously, well see, just watching that video, I was interested, because it’s speaking in my language and it’s a cartoon with people that look like me, they’re speaking my language and they’re talking about things that are happening with me, I’m going to definitely listen to that T4DC Pg 23-24 - … but we think we have the first experience being targeted, we don't feel all right, we don't feel all right about being targeted, we thought we are Australians, we are to be treated as Australian - - - Pg 66 SD2 - Because we have this language barrier, even with our own kids. We don’t speak to them. If we want to convey anything it has to be in English. English is the main language for them. For someone to give it to them in an easy way, it should be one of them. It should be one of them, and that is how we tackle, how we give out information and for people to answer them, we talk either in our language or general and all that T4 SMC Pg10 - The generation gap is very, very now. It is very coming. We the elders who came, we are living with our children but we have the youth leaders. The youth leaders are there. When we call them, the youth leaders are coming, they come to us because they are still good people. Yeah. So, they come from the community then call the youth and the youth whenever they call them, they come together. T4 SMC Pg10 |
